# Supplementary material for: Design for improving corrosion resistance of duplex stainless steels by wrapping inclusions with niobium armour
Source: Nat Commun. 2023 Nov 30;14:7869. doi: 10.1038/s41467-023-43752-8 (PMC10689471; doi:10.1038/s41467-023-43752-8)
Supplement: Supplementary file 1 — Supplementary Information [file 41467_2023_43752_MOESM1_ESM.pdf]

Supplementary Information for:

Design for improving corrosion resistance of duplex stainless steels by wrapping inclusions with niobium armour

Shucai Zhang[1], Hao Feng[1], Huabing Li[1,2]*, Zhouhua Jiang[1], Tao Zhang[3], Hongchun Zhu[1], Yue Lin[1], Wei Zhang[4,5], Guoping Li[6,7]

[1]School of Metallurgy, Northeastern University, Shenyang, 110819, China

[2]Key Laboratory for Ecological Metallurgy of Multimetallic Ores (Ministry of Education), Northeastern University, Shenyang, 110819, China

[3]School of Materials Science and Engineering, Northeastern University, Shenyang, 110819, China

[4]Central Iron and Steel Research Institute, Beijing, 100081, China

[5]CITIC Metal Co., Ltd., Beijing, 100027, China

[6]Shanxi Taigang Stainless Steel Co., Ltd., Taiyuan, 030003, China

[7]State Key Laboratory of Advanced Stainless Steel Materials, Taiyuan, 030003, China

*Corresponding author

E-mail: lihb@smm.neu.edu.cn (Huabing Li)

This file include:

Supplementary Figures 1 to 16

Supplementary Note 1

Supplementary Tables 1 to 8

Supplementary References 1 to 2

Supplementary Figures

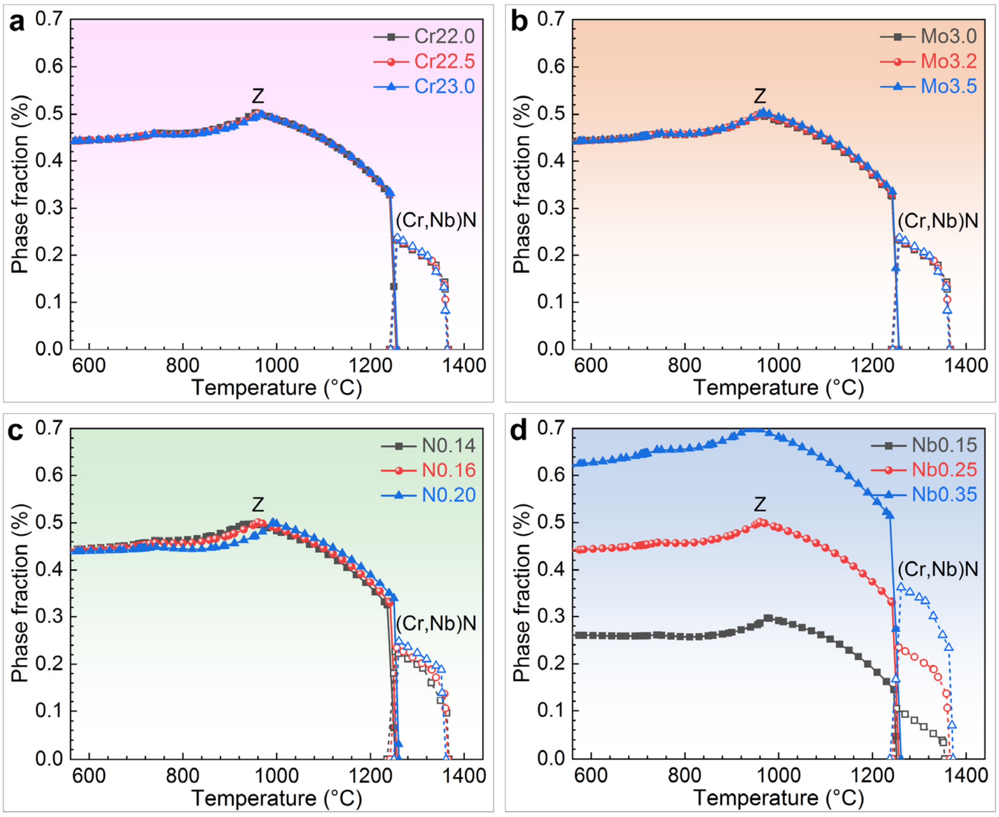

Supplementary Fig. 1 Effect of key elements content on the precipitation behavior of Nb-bearing phases in S32205 DSSs: a Cr, **b** Mo, **c** N, and **d** Nb. Source data are provided as a Source Data file.

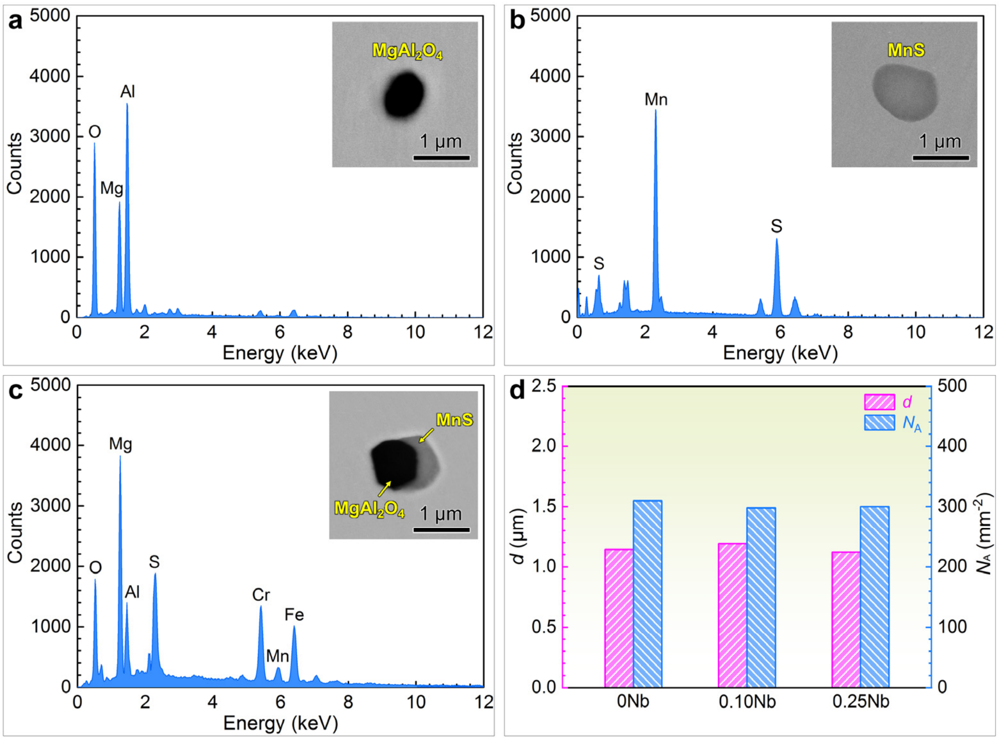

Supplementary Fig. 2 Characterization of the inclusions. Morphologies and compositions of typical inclusions in S32205 DSSs: **a** $MgAl_2O_4$, **b** MnS, and **c** $MgAl_2O_4$-MnS. **d** Average equivalent diameter (*d*) and number density (N_A) of the inclusions. Source data are provided as a Source Data file.

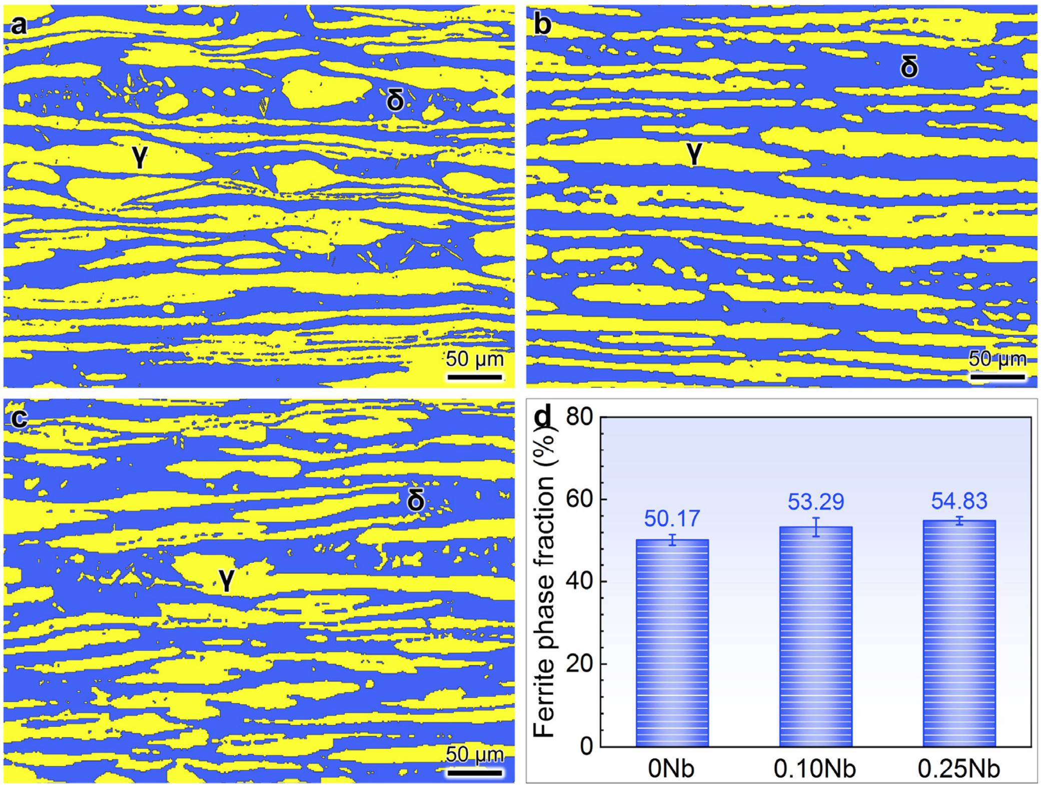

Supplementary Fig. 3 Characterization of heat-treated microstructures. Typical microstructure and ferrite phase fractions of S32205 DSSs solution treated at 1050 °C for 0.5 h: **a** 0Nb, **b** 0.10Nb, **c** 0.25Nb, and **d** ferrite phase fractions. γ and δ are austenite phase and ferrite phase, respectively. All the error bars in (**d**) represent the standard deviation of at least thirty independent measurements. Source data are provided as a Source Data file.

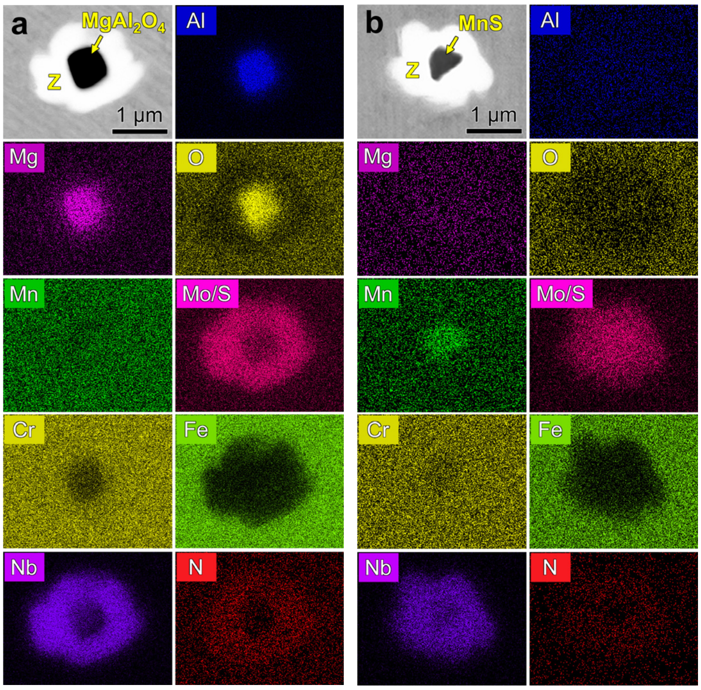

Supplementary Fig. 4 Characterization of the inclusion@Z core-shell structures. EDS elemental mappings of the inclusion@Z core-shell structures in solution-treated 0.25Nb S32205 DSS: **a** $MgAl_2O_4$@Z and **b** MnS@Z core-shell structures.

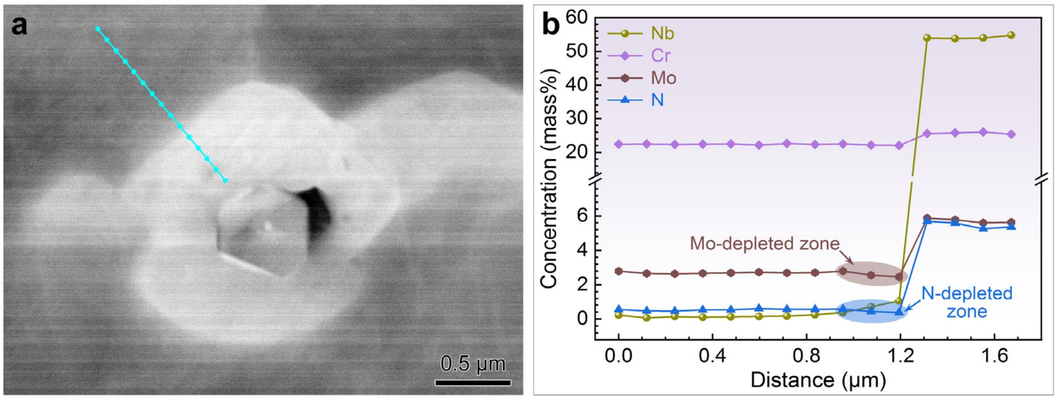

Supplementary Fig. 5 STEM and EDS characterization of the Z phase. **a** STEM dark-field image of the Z phase, and **b** STEM-EDS line profile analysis across the Z phase. Source data are provided as a Source Data file.

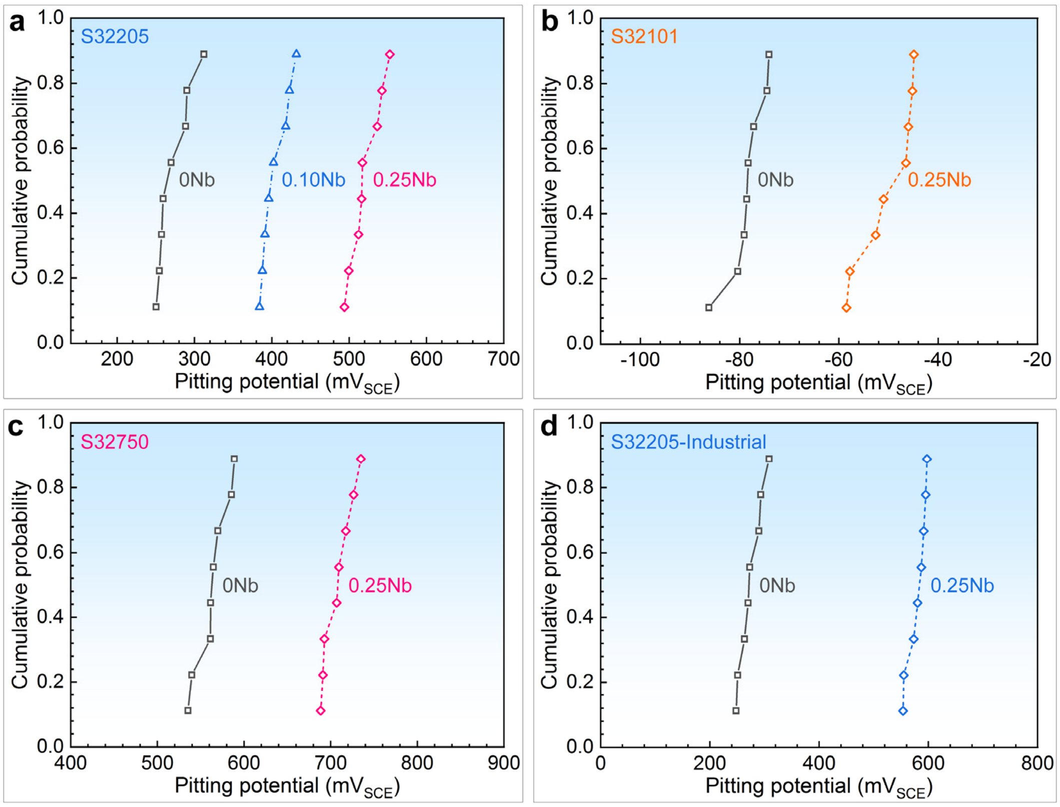

Supplementary Fig. 6 Cumulative probability distribution of pitting potential. Cumulative probability distribution of pitting potential of various Nb-free and Nb-bearing DSSs in double-concentration simulated seawater at 72 °C (pH 8.2): **a** S32205, **b** S32101, **c** S32750, and **d** S32205-Industrial. Source data are provided as a Source Data file.

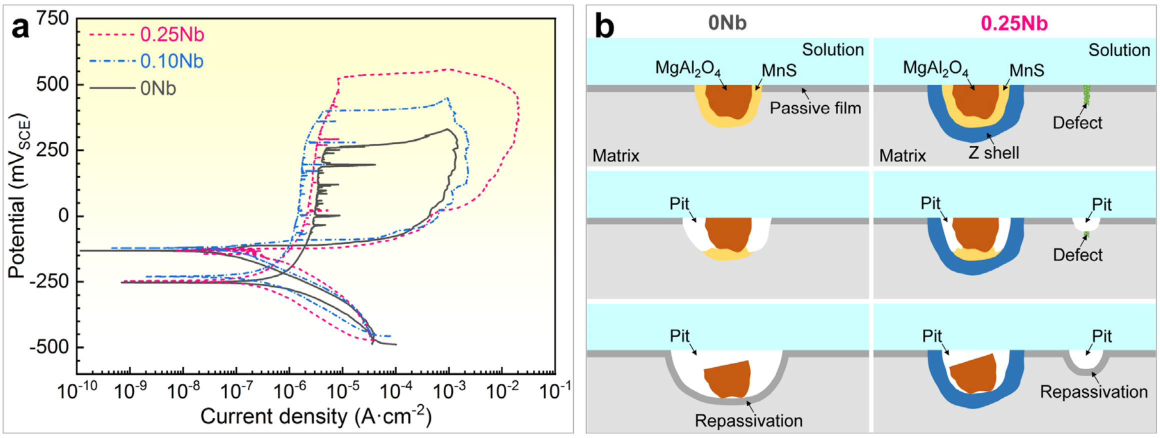

Supplementary Fig. 7 Repassivation behaviour of S32205 DSSs. a Cyclic polarization curves in double-concentration simulated seawater at 72 °C (pH 8.2), and **b** Schematic diagram of corrosion and repassivation process. Source data are provided as a Source Data file.

Supplementary Note 1

The Gumbel extreme value distribution is used to explore the effect of Nb microalloying on pit growth [1]. The depths of 15 deepest pits were arranged by small to large order, and the probability of pit depths $F(Y)$ can be calculated by [2]:

$$F(Y) = 1 - \frac{n}{N+1} \tag{1}$$

where n is the rank in the ordered pit depth, and N is the total number of selected pits. The reduced variant (Y) can be calculated by [2]:

$$Y = -\ln\{-\ln[F(Y)]\} \tag{2}$$

The maximum pit depth can be calculated by the Gumbel type extreme value distribution expressed in the following form [2]:

$$Pit_{max} = \mu + \alpha \ln T \tag{3}$$

$$T = S/s \tag{4}$$

where Pit_{max} is the maximum pit depth, μ is the central parameter (the most frequent value), and α is the scale parameter that defines the width of the distribution. S is the area over which a prediction is to be made, s is the statistical area of the sample, and T is the ratio of predicted area to statistical area.

Supplementary Fig. 8a and b show the cumulative probability of pit depths and the

Gumbel probability plots of S32205 DSSs after immersion corrosion in a 6% $FeCl_3$ solution at 50 °C for 12 h. The proportion of inclusions wrapped by the Z phase in 0.10Nb and 0.25Nb steels are 49.3% and 75.8% (**Fig. 2**e), indicating that the effective areas (s) used for statistics in 0.10Nb and 0.25Nb steels are 50.7% and 24.2% of that in 0Nb steel, respectively. Therefore, the Gumbel distribution parameters (μ = 131.6 and α = 46.5) and the maximum pit depth (243.5 μm) of 0Nb steel, together with the area proportions of 0.10Nb and 0.25Nb steels are used to predict the maximum pit depth of 0.10Nb and 0.25Nb steels, as follows:

$$Pit_{\max}^{0\mathrm{Nb}} = 131.6+46.5\ln T \quad (5)$$

$$Pit_{\max}^{0.10\mathrm{Nb}} = 131.6+46.5\ln(0.507T) \quad (6)$$

$$Pit_{\max}^{0.25\mathrm{Nb}} = 131.6+46.5\ln(0.242T) \quad (7)$$

Supplementary Fig. 8c illustrates that the predicted values for the maximum pit depth of 0.10Nb and 0.25Nb steels are 211.9 and 177.5 μm, respectively, which are close to the measured values (192.5 and 164.0 μm). The errors for 0.10Nb and 0.25Nb steels are 9.1% and 7.6%, respectively. In summary, the pit growth of the three S32205 DSSs can be well modelled by the Gumbel extreme value distribution. Once the inclusions are wrapped by the Z phase, the proportion of inclusions that may induce deep pits is considerably decreased, therefore, the maximum pit depth of Nb-bearing steels becomes significantly smaller.

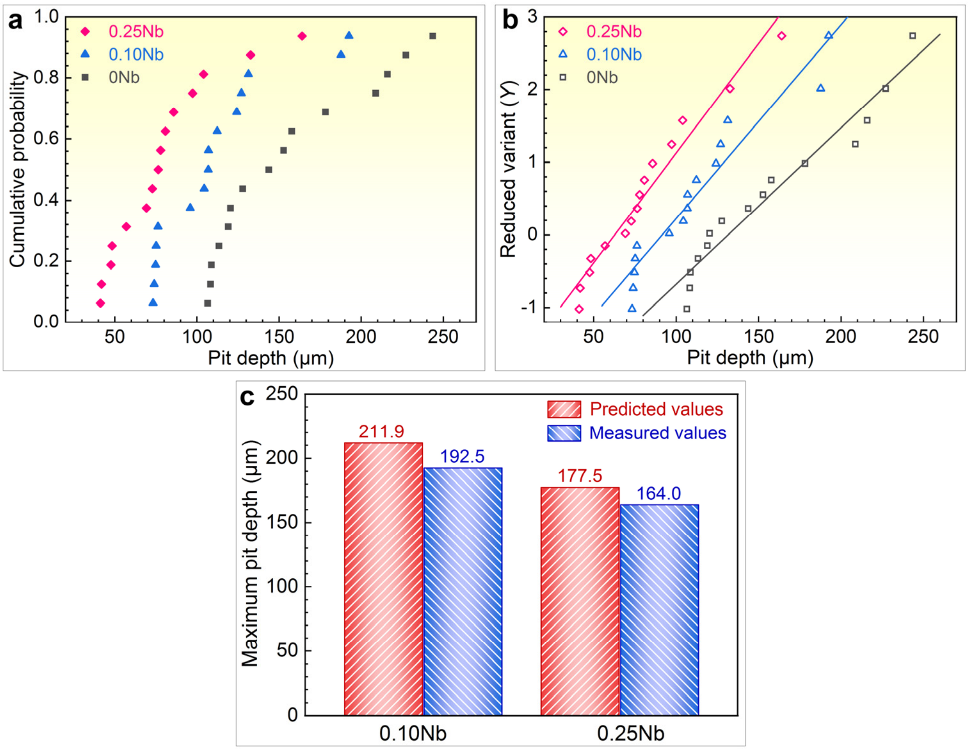

Supplementary Fig. 8 Gumbel extreme value distribution. **a** Cumulative probability of pit depths and **b** the Gumbel probability plots of S32205 DSSs after immersion corrosion in a 6% $FeCl_3$ solution at 50 °C for 12 h. **c** Comparison between the predicted and measured values for the maximum pit depth of 0.10Nb and 0.25Nb steels. Source data are provided as a Source Data file.

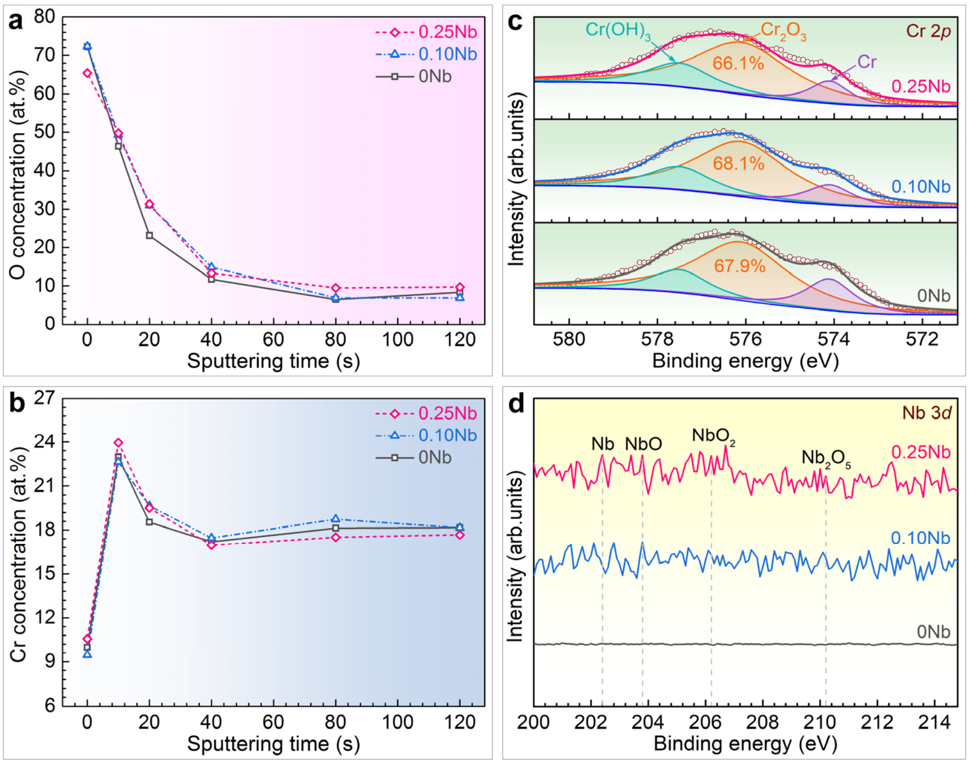

Supplementary Fig. 9 Characterization of passive films. Compositions of passive films formed on S32205 DSSs: **a** O concentration, **b** Cr concentration, **c** XPS spectra of Cr, and **d** XPS spectra of Nb. Source data are provided as a Source Data file.

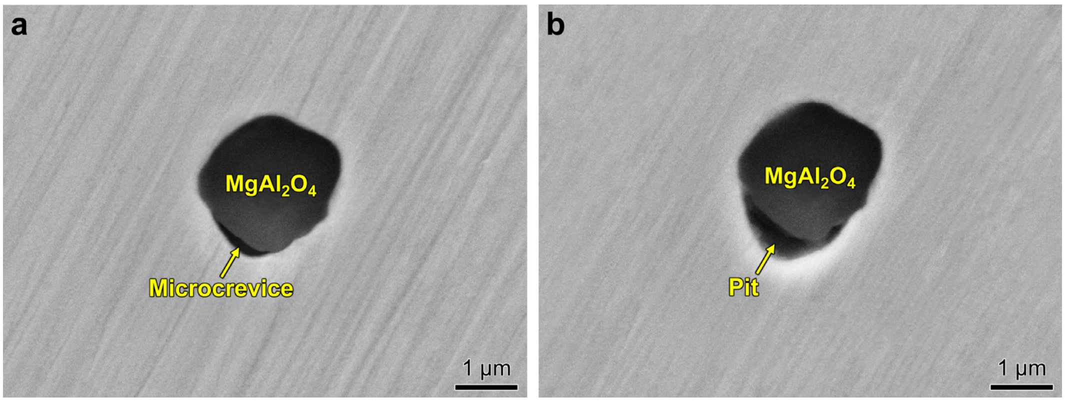

Supplementary Fig. 10 Characterization of 0Nb S32205 DSS before and after immersion corrosion. SEM morphologies of $MgAl_2O_4$ inclusion in the steel before (**a**) and after (**b**) immersion corrosion in a 6% $FeCl_3$ solution at 50 °C for 12 h.

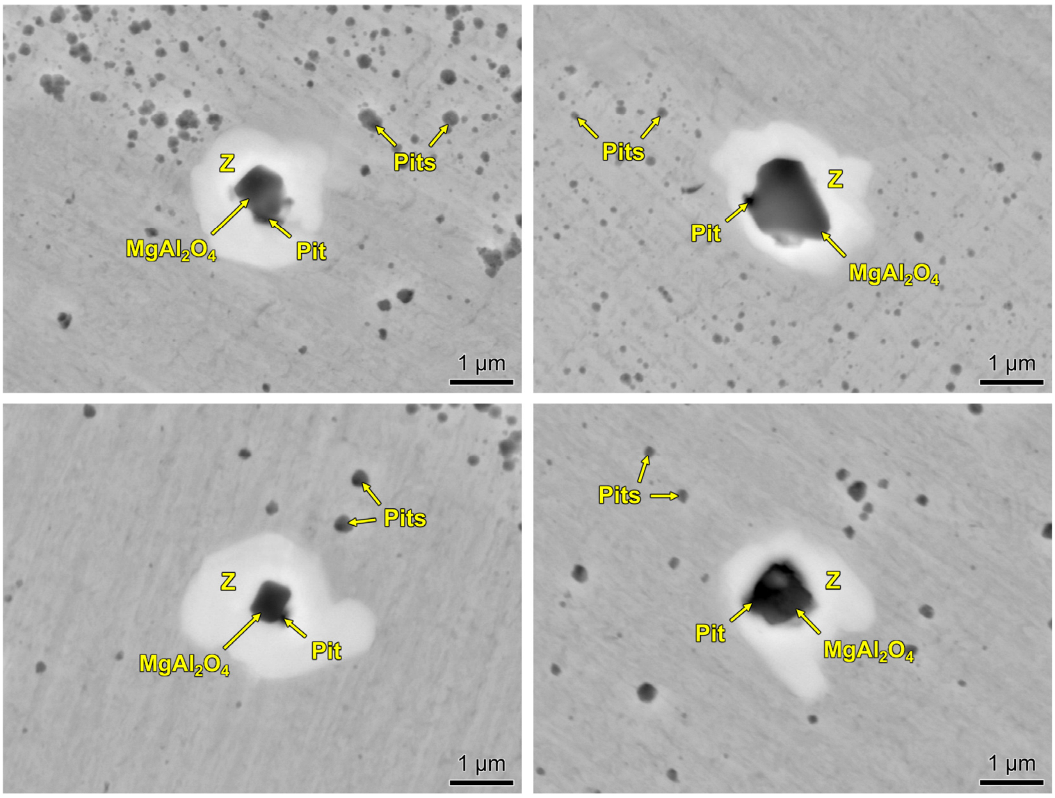

Supplementary Fig. 11 Characterization of 0.25Nb S32205 DSS after immersion corrosion. SEM morphologies of the inclusion@Z core-shell structure in the steel after immersion corrosion in a 6% $FeCl_3$ solution at 50 °C for 10 d.

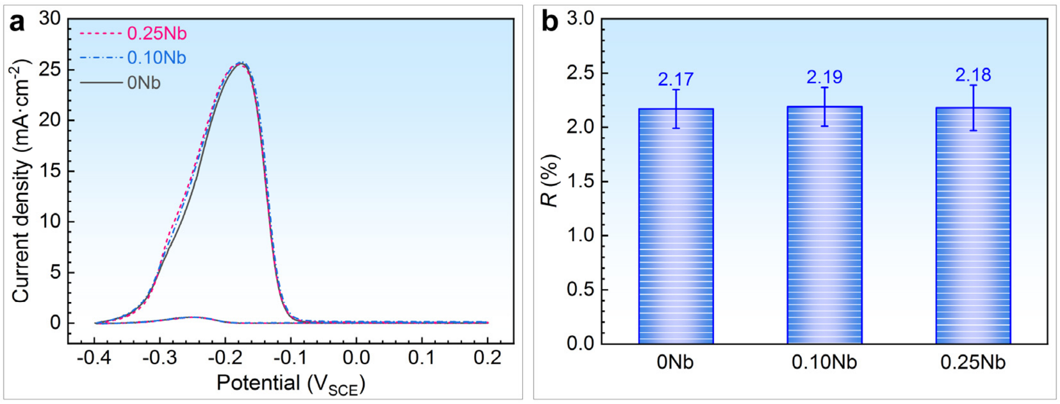

Supplementary Fig. 12 Double loop electrochemical potentiokinetic reactivation (DL-EPR) results of S32205 DSSs. a Typical DL-EPR curves, and **b** Degree of sensitization (R value). R=(I_r/I_a)×100, I_a and I_r represent the peak activation current density and peak reactivation current density measured during the forward and reverse scans, respectively. All the error bars in (**b**) represent the standard deviation (n = 8 independent experiments). Source data are provided as a Source Data file.

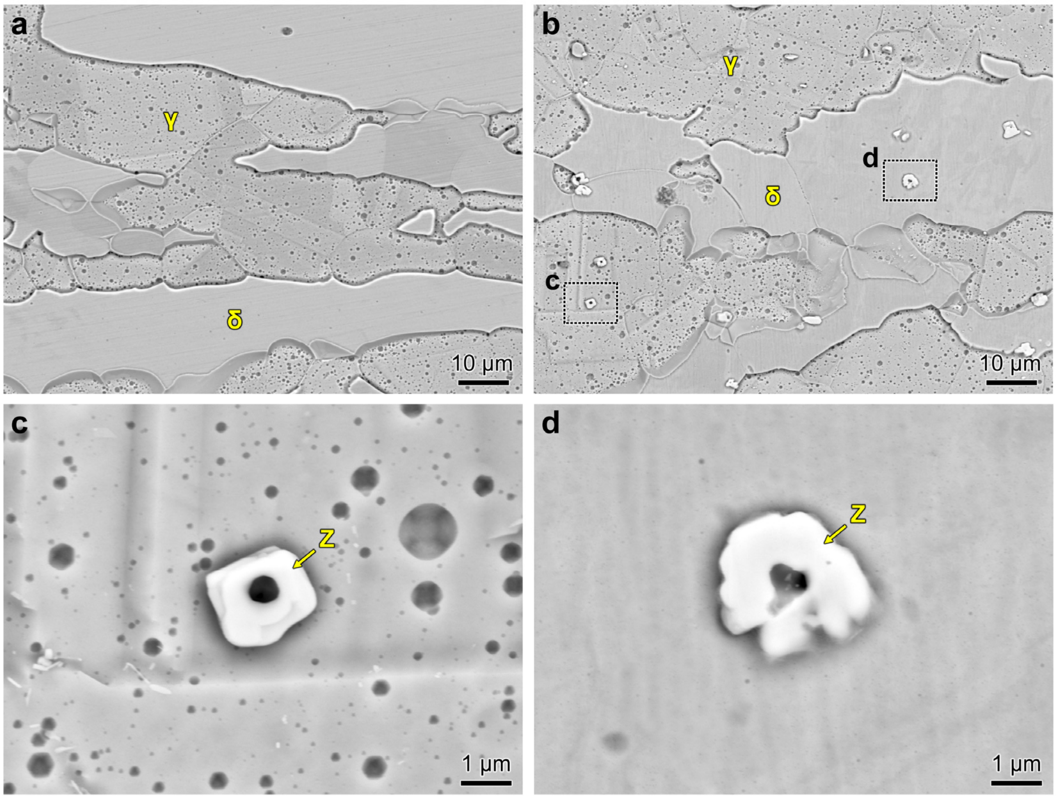

Supplementary Fig. 13 IGC morphologies of S32205 DSSs after DL-EPR tests. a 0Nb, **b** 0.25Nb, **c** and **d** Enlarged views of the regions circled by the dashed boxes in (**b**). γ and δ are austenite phase and ferrite phase, respectively.

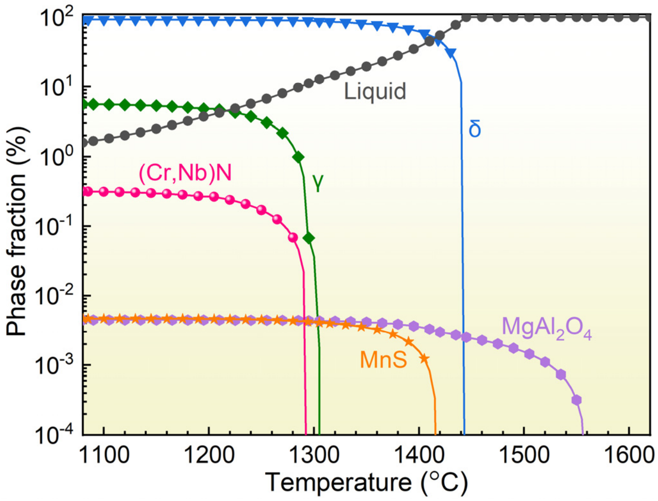

Supplementary Fig. 14 Nonequilibrium solidification process of 0.25Nb S32205 DSS calculated by the Gulliver-Scheil model in FactSage software. γ and δ are austenite phase and ferrite phase, respectively. Source data are provided as a Source Data file.

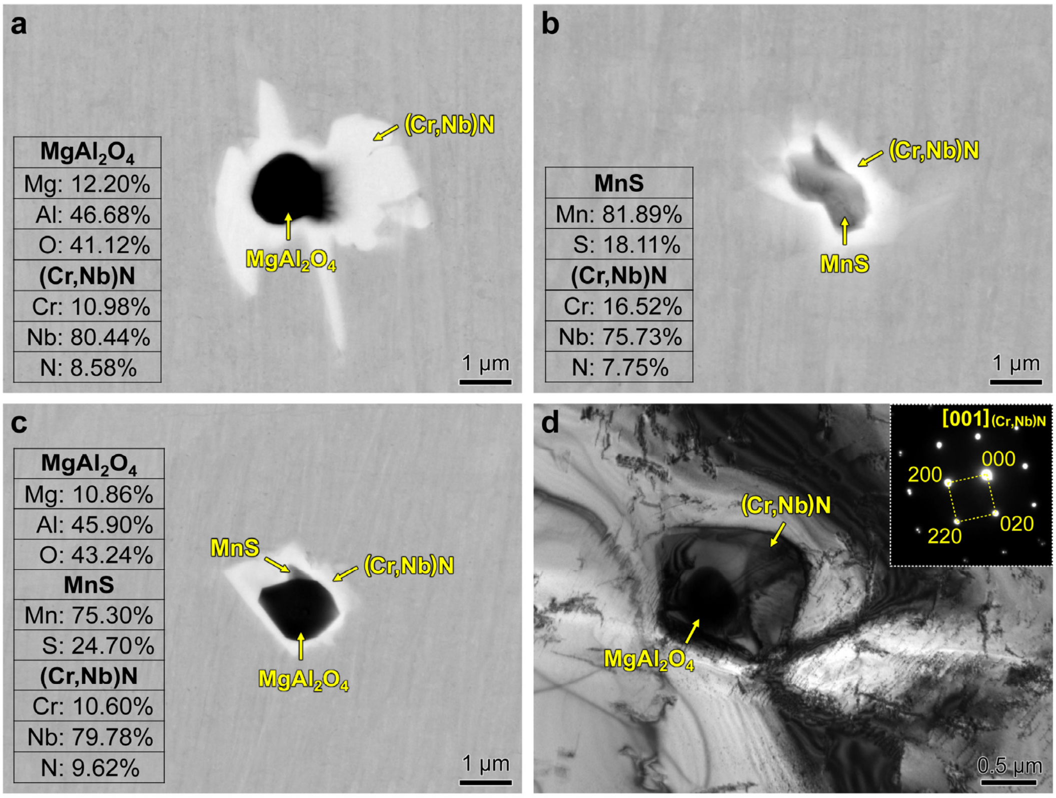

Supplementary Fig. 15 Characterization of the inclusion@(Cr,Nb)N core-shell structures. Morphologies and SEM-EDS compositions (wt.%) of the inclusion@(Cr,Nb)N core-shell structures in as-cast 0.25Nb S32205 DSS: **a** $MgAl_2O_4$@(Cr,Nb)N, **b** MnS@(Cr,Nb)N, and **c** $MgAl_2O_4$-MnS@(Cr,Nb)N core-shell structures. **d** TEM bright-field image and corresponding selected area electron diffraction pattern of (Cr,Nb)N.

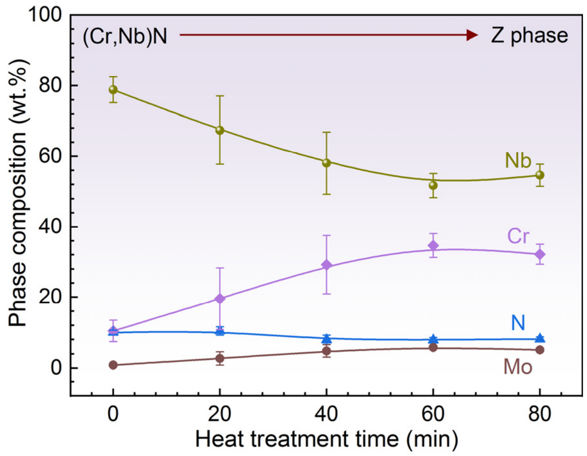

Supplementary Fig. 16 Chemical composition variation of Nb-bearing phase in as-cast 0.25Nb S32205 DSS after heat treatment at 1180 °C for different times. All the error bars represent the standard deviation of at least seven independent measurements. Source data are provided as a Source Data file.

Supplementary Tables

Supplementary Table 1 Chemical compositions (wt.%) of microalloyed S32205 DSSs used for Thermo-Calc calculations.

Steels	C	Si	Mn	Cr	Ni	Mo	N	Ti	V	Nb	Fe
Ti0.25	0.02	0.5	1.2	22.5	5.5	3.0	0.16	0.25	–	–	Bal.
V0.25	0.02	0.5	1.2	22.5	5.5	3.0	0.16	–	0.25	–	Bal.
Nb0.25	0.02	0.5	1.2	22.5	5.5	3.0	0.16	–	–	0.25	Bal.
Cr22.0	0.02	0.5	1.2	22.0	5.5	3.0	0.16	–	–	0.25	Bal.
Cr23.0	0.02	0.5	1.2	23.0	5.5	3.0	0.16	–	–	0.25	Bal.
Mo3.2	0.02	0.5	1.2	22.5	5.5	3.2	0.16	–	–	0.25	Bal.
Mo3.5	0.02	0.5	1.2	22.5	5.5	3.5	0.16	–	–	0.25	Bal.
N0.14	0.02	0.5	1.2	22.5	5.5	3.0	0.14	–	–	0.25	Bal.
N0.20	0.02	0.5	1.2	22.5	5.5	3.0	0.20	–	–	0.25	Bal.
Nb0.15	0.02	0.5	1.2	22.5	5.5	3.0	0.16	–	–	0.15	Bal.
Nb0.35	0.02	0.5	1.2	22.5	5.5	3.0	0.16	–	–	0.35	Bal.

Note: The chemical compositions (wt.%) of Cr22.5, Mo3.0, and N0.16 are same with that of Nb0.25.

Supplementary Table 2 Lattice disregistries between Nb-bearing phases and typical inclusions.

Nucleation pairs	Matching planes	Matching directions		Interatomic spacing (nm)		θ (°)	Lattice disregistry (%)
		$[uvw]_s$	$[uvw]_n$	$d[uvw]_s$	$d[uvw]_n$		
$MgAl_2O_4$//(Cr,Nb)N	$(001)_{MgAl2O4}//(111)_{(Cr,Nb)N}$	$[110]$	$[\bar{1}\bar{1}2]$	1.142	1.076	0	5.61
		$[310]$	$[\bar{1}01]$	1.277	1.242	3.43	
		$[1\bar{1}0]$	$[\bar{1}10]$	0.571	0.621	0	
	$(111)_{MgAl2O4}//(111)_{(Cr,Nb)N}$	$[\bar{1}10]$	$[\bar{1}10]$	0.571	0.621	0	8.06
		$[\bar{2}11]$	$[\bar{2}11]$	0.989	1.076	0	

		$[\bar{1}01]$	$[\bar{1}01]$	0.571	0.621	0	
MnS//(Cr,Nb)N	$(111)_{MnS}//(110)_{(Cr,Nb)N}$	$[\bar{1}12]$	$[1\bar{1}0]$	0.642	0.621	0	7.30
		$[\bar{2}12]$	$[1\bar{1}1]$	0.741	0.761	5.26	
		$[\bar{1}10]$	$[001]$	0.371	0.439	0	
	$(110)_{MnS}//(111)_{(Cr,Nb)N}$	$[001]$	$[\bar{1}\bar{1}2]$	0.524	0.539	0	8.34
		$[\bar{1}12]$	$[\bar{2}12]$	0.642	0.621	5.26	
		$[\bar{1}10]$	$[\bar{1}10]$	0.371	0.311	0	
$MgAl_2O_4$//Z	$(100)_{MgAl2O4}//(110)_Z$	$[010]$	$[001]$	0.808	0.739	0	5.26
		$[011]$	$[\bar{2}21]$	1.142	1.132	4.29	
		$[001]$	$[\bar{2}20]$	0.808	0.858	0	
	$(110)_{MgAl2O4}//(011)_Z$	$[001]$	$[0\bar{1}1]$	0.808	0.799	0	2.87
		$[\bar{1}12]$	$[1\bar{1}1]$	0.989	1.004	1.98	
		$[\bar{1}10]$	$[100]$	0.571	0.607	0	
MnS//Z	$(100)_{MnS}//(110)_Z$	$[011]$	$[001]$	0.741	0.739	0	5.67
		$[031]$	$[\bar{1}11]$	0.828	0.855	3.57	
		$[010]$	$[\bar{1}10]$	0.371	0.429	0	
	$(100)_{MnS}//(111)_Z$	$[011]$	$[110]$	0.741	0.744	0	5.86
		$[031]$	$[\bar{1}\bar{1}2]$	0.828	0.858	3.43	
		$[010]$	$[1\bar{1}0]$	0.371	0.429	0	

Supplementary Table 3 Chemical compositions (wt.%) of experimental S32205 DSSs with various Nb contents.

Steels	C	Si	Mn	P	S	Cr	Ni	Mo	N	Nb	Fe
0Nb	0.018	0.49	1.21	0.005	0.0026	22.51	5.62	3.12	0.16	0	Bal.
0.10Nb	0.017	0.48	1.19	0.005	0.0028	22.50	5.62	3.09	0.16	0.10	Bal.
0.25Nb	0.015	0.50	1.20	0.005	0.0029	22.52	5.59	3.13	0.16	0.25	Bal.

Supplementary Table 4 Calculation results of lattice misfits and interfacial energies.

Nucleation pairs	Matching planes	n	α (°)	δ	|b| (nm)	σ ($J\cdot m^2$)	$\overline{\sigma}$ ($J\cdot m^2$)
$MgAl_2O_4$//Z	$(400)_{MgAl2O4}//(110)_Z$	1	6.85	δ_1=0.081	$\|b\|_1$=0.212	σ_1=0.419	0.419
	$(040)_{MgAl2O4}//(1\bar{1}0)_Z$	1	6.85	δ_2=0.081	$\|b\|_2$=0.212	σ_2=0.419	
Ferrite//Z	$(110)_{Ferrite}//(110)_Z$	1	4.15	δ_1=0.035	$\|b\|_1$=0.209	σ_1=0.248	0.349
	$(1\bar{1}0)_{Ferrite}//(102)_Z$	2	35.19	δ_2=0.145	$\|b\|_2$=0.226	σ_2=0.593	

Supplementary Table 5 Chemical compositions (wt.%) of DSSs used for Thermo-Calc calculations.

Types	Steels	C	Si	Mn	Cr	Ni	Mo	Cu	Co	N	Nb	Fe
Lean	S32101	0.016	0.58	5.02	21.50	1.51	0.32	0.49	–	0.22	0.25	Bal.
	S32304	0.015	0.53	1.46	23.12	4.55	0.33	0.44	–	0.12	0.25	Bal.
Standard	S32205	0.015	0.50	1.20	22.52	5.59	3.13	–	–	0.16	0.25	Bal.
Super	S32750	0.016	0.52	0.71	25.05	7.02	4.01	–	–	0.28	0.25	Bal.
Hyper	S32707	0.014	0.48	1.12	26.86	7.08	4.63	0.95	0.97	0.41	0.25	Bal.

Supplementary Table 6 Chemical compositions (wt.%) of experimental S32101 and S32750 DSSs.

Steels	C	Si	Mn	Cr	Ni	Mo	Cu	N	Nb	Fe
S32101-0Nb	0.018	0.62	5.06	21.53	1.48	0.29	0.51	0.22	0	Bal.
S32101-0.25Nb	0.016	0.58	5.02	21.50	1.51	0.32	0.49	0.22	0.25	Bal.
S32750-0Nb	0.015	0.49	0.72	25.03	7.06	4.08	–	0.28	0	Bal.
S32750-0.25Nb	0.016	0.52	0.71	25.05	7.02	4.01	–	0.28	0.25	Bal.

Supplementary Table 7 Room-temperature tensile properties of S32205 DSSs with various Nb contents.

Steels	Yield strength (MPa)	Ultimate tensile strength (MPa)	Elongation (%)
0Nb	514.8±9.0	750.9±1.9	37.7±2.4
0.10Nb	572.6±15.3	779.1±8.1	36.7±1.4
0.25Nb	541.7±19.8	768.5±7.2	35.8±1.9

Supplementary Table 8 Nanohardness and elastic modulus of the steel matrix and the Z phase measured by nanoindenter.

Phases	Nanohardness (GPa)	Elastic modulus (GPa)
Austenite (γ)	6.37±1.01	192.70±16.97
Ferrite (δ)	5.68±0.73	207.97±13.11
Z phase	7.52±1.12	237.99±34.74

Supplementary References

[1] Valor, A., Caleyo, F., Alfonso, L., Rivas, D. & Hallen, J. M. Stochastic modeling of pitting corrosion: A new model for initiation and growth of multiple corrosion pits, *Corros. Sci.* **49**, 559–579 (2007).

[2] Zhang, T. et al. Corrosion of pure magnesium under thin electrolyte layers, *Electrochim. Acta* **53**, 7921–7931 (2008).
